# LncRNA HOTAIR-mediated Wnt/β-catenin network modeling to predict and validate therapeutic targets for cartilage damage

**DOI:** 10.1186/s12859-019-2981-4

**Published:** 2019-07-31

**Authors:** Wei Zhou, Xiaojuan He, Ziyi Chen, Danping Fan, Yonghua Wang, Hui Feng, Ge Zhang, Aiping Lu, Lianbo Xiao

**Affiliations:** 1 Institute of Arthritis Research, Shanghai Academy of Chinese Medical Sciences, Guanghua Integrative Medicine Hospital / Shanghai University of T.C.M, Shanghai, 200052 China; 2 Institute of Basic Research in Clinical Medicine, China Academy of Chinese Medical Sciences, Beijing, 100700 China; 3 Department of Allergy, The Third Affiliated Hospital of Shenzhen University, Shenzhen, 518020 China; 4 Musculoskeletal Research Laboratory, Department of Orthopaedics & Traumatology, Faculty of Medicine, The Chinese University of Hong Kong, Shatin, Hong Kong, 999077 SAR China; 5 College of Life Science, Northwest University, Xi’an, 710000 Shaanxi China; 6 Institute of Integrated Bioinformedicine & Translational Science, School of Chinese Medicine, Hong Kong Baptist University, Hong Kong, China

**Keywords:** LncRNA HOTAIR, Dynamic network, Therapeutic targets, Dynamic mechanism, Cartilage damage

## Abstract

**Background:**

Cartilage damage is a crucial feature involved in several pathological conditions characterized by joint disorders, such as osteoarthritis and rheumatoid arthritis. Accumulated evidences showed that Wnt/β-catenin pathway plays a role in the pathogenesis of cartilage damage. In addition, it is experimentally documented that lncRNA (long non-coding RNA) HOTAIR plays a key role in the regulation of Wnt/β-catenin pathway based on directly decreased WIF-1 expression. Further, it is reported that Wnt/β-catenin pathway is a potent pathway to regulate the expression of MMP-13, which is responsible for degradation of collagen type II in articular cartilage. It is increasingly recognized that systems modeling approach provides an opportunity to understand the complex relationships and direct quantitative analysis of dynamic network in various diseases.

**Results:**

A dynamic network of lncRNA HOTAIR-mediated Wnt/β-catenin pathway regulating MMP-13 is developed to investigate the dynamic mechanism of the network involved in the pathogenesis of cartilage damage. Based on the network modeling, the potential therapeutic intervention point Axin is predicted and confirmed by the experimental validation.

**Conclusions:**

Our study provides a promising strategy for revealing potential dynamic mechanism and assessing potential targets which contribute to the prevention of the pathological conditions related to cartilage damage.

## Background

Cartilage damage is a central feature in joint diseases that is common in the patients with osteoarthritis (OA) and rheumatoid arthritis (RA) [1]. Evidences to date indicates that cartilage damage has still been a great challenge in clinical treatment [2]. Cartilage is maintained by chondrocytes that secrete the extracellular matrix (ECM) components, such as collagen and aggrecan [3]. Cartilage ECM is thought to be regulated by matrix metalloproteinases (MMPs) and aggrecanases, two major mediators in response to the breakdown of extracellular cartilage matrix. In particular, MMP-13 is considered to be a key enzyme involved in collagen degradation in articular cartilage, while increased production of MMP-13 by chondrocytes has been related to cartilage degradation [4]. Loss of aggrecan from cartilage is thought to be a reversible process whilst the collagen degradation is believed to be irreversible, contributing to joint deformity and functional disability [5, 6]. It seems that the prevention of collagen degradation is the key to develop effective therapies for cartilage damage. Although accumulated evidences suggested that many factors have provided crucial understanding on the complex mechanisms underlying cartilage damage [7, 8]. However, cartilage repair seems limited or occurs only infrequently during the current treatment with methotrexate and TNF inhibitor [2]. The molecular mechanisms involved in disease initiation and progression are still incompletely understood, since cartilage damage is a complex pathological condition that often involves various interactions between genes, pathways and small RNAs [9].

Investigation of potential molecular mechanisms of cartilage development and degeneration will offer important references for the treatment of related joint diseases. Increasing evidences indicate several crucial molecular pathways in cartilage damage, one of which is Wnt/β-catenin signaling pathway [10]. The Wnt/β-catenin signaling pathway is consisted of a family of conserved secreted signaling molecules which plays a vital role in the regulation of chondrocyte proliferation, differentiation, and apoptosis. Wnt signaling is initiated by targeting the “destruction complex” consisting of the core scaffolding proteins Axin and adenomatous polyposis coli (APC), in complex with glycogen synthase kinase 3 (GSK3) that promotes the phosphorylation of β-catenin. In the absence of Wnt ligands, phosphorylated β-catenin is assigned to subsequent ubiquitylation and proteasomal degradation [11]. Upon Wnt activation, the Wnt proteins bind to its receptors resulting in the dissociation of the destruction complex and accumulation of cytoplasmic β-catenin. Consequently, stabilised β-catenin translocates to the nucleus and functions as a co-factor of T cell factor/lymphoid enhancer-binding factor (TCF/LEF) transcription factors to trigger the transcription of Wnt target genes [11, 12]. It is reported that activation of Wnt/β-catenin signaling drives rapid gene expression of MMPs, suggesting that this pathway plays a key role in chondrocytes’ regulatory machinery releated to the degradation of cartilage matrix [13]. Given the fact that Wnt/β-catenin signaling is a complex network, more attention should be paid to the Wnt family components as molecular targets for specific targeted therapy in the treatment of cartilage damage.

In recent years, increasing evidence suggests that long non-coding RNAs (lncRNAs) play important roles in the regulation of a variety of biological processes [14, 15]. It has been demonstrated that altered levels of lncRNAs could lead to aberrant gene expression that relates to various disease states and biological functions [16–18]. The recent study demonstrated that HOTAIR as a widely focused lncRNA is a potential biomarker contributing to the RA pathogenesis [19]. HOTAIR is reported to be involved in the regulation of the Wnt/β-catenin signaling pathway based on directly decreased WIF-1 expression through promoting its histone H3K27 methylation in the promoter region [20]. Moreover, Wnt/β-catenin signaling was identified as a potent pathway to activate MMP-13 expression in chondrocytes [21]. Taken all together, a hypothesis is raised that HOTAIR activates MMP-13 expression to block cartilage damage through regulating Wnt/β-catenin signaling pathway. Although genetic evidence points to important roles of HOTAIR in regulating RA [19], and the key processes occurring in HOTAIR-regulated cartilage damage have already been established [19–21], understanding the details of the kinetic interplay between HOTAIR and key mediators involved in cartilage damage, especially the overall dynamic characteristic and precise regulation mechanisms of HOTAIR-mediated Wnt/β-catenin signaling pathway contributions to the degradation of ECM and the pathogenesis of cartilage damage are still mysterious. Thus it is an urgent need for a well-established therapeutic or controlling method for further understanding the overall dynamic molecular mechanism of cartilage damage so as to develop novel therapeutic interventions for the treatment of cartilage damage.

Fortunately, it has become increasingly recognized that systems modeling method offers a chance to understand the potential dynamic mechanism and direct quantitative analysis of signaling pathway in various biological processes [22, 23]. It provides an opportunity to understand direct quantitative analysis of dynamic pathway, and shows directly the effects of changes caused by multi parameters in the dynamic behavior of network model. The mathematical modeling of pathways was developed by employing a chemical kinetic reactions approach based on ordinary differential equations (ODEs) [24]. An ODE model is more detailed than other simple models (such as boolean model), since it presupposes that molecular concentrations are sufficiently large so as to be approximated with a continuous and deterministic description [25]. The modeling of chemical reactions can be achieved either by using differential equations built based on the law of mass action or by the use of their stochastic counterpart [26, 27]. In the previous study, we developed a dynamic model of miRNA-mediated mammalian circadian clock system and found that the amplitude and frequency of the oscillation could be significantly altered through the miR-206-mediated control [28]. Therefore, in this study, we attempt to establish a detailed quantitative mathematical model for cartilage damage, which involves the lncRNA HOTAIR-mediated Wnt/β-catenin signaling pathway related to the degradation of ECM and the pathogenesis of cartilage damage. The model and related discovery will not only be helpful for deep understanding potential dynamic molecular mechanism of lncRNA HOTAIR-mediated Wnt/β-catenin signaling pathway, but also provide a new view to predict potential therapeutic targets for further validation by experiment which contribute to the prevention of the pathological conditions that related to cartilage damage.

## Results

### Dynamics of HOTAIR-mediated Wnt/β-catenin signaling pathway

The Wnt/β-catenin signaling pathway has crucial roles in essential cellular processes such as cell growth, proliferation and apoptosis. Dysfunction of Wnt/β-catenin signaling pathway is involved in a host of diseases including cartilage damage in RA [10, 29]. In addition, it has become increasingly evident that lncRNAs may be involved in the regulation of various cellular and molecular pathways including Wnt/β-catenin signaling pathway. Despite of many molecular advances, the pathway dynamics remain not well interpreted. Since the general description of the Wnt/β-catenin signaling pathway does not consider the concentrations of signaling molecules and the quantitative description of pathway’s dynamic behavior. As the accumulation of quantitative data becomes more important for understanding the potential dynamic mechanism of main components in the signaling pathway, the development of theoretical models will be able to serve as test beds for evaluating hypotheses, describing current knowledge and developing novel predictions. Therefore, in this work, a model of HOTAIR-mediated Wnt/β-catenin pathway was developed with the aim of elucidating potential dynamic mechanism, predicting function as well as investigating key pathway components that play roles in cartilage damage.

**Fig. 1:**
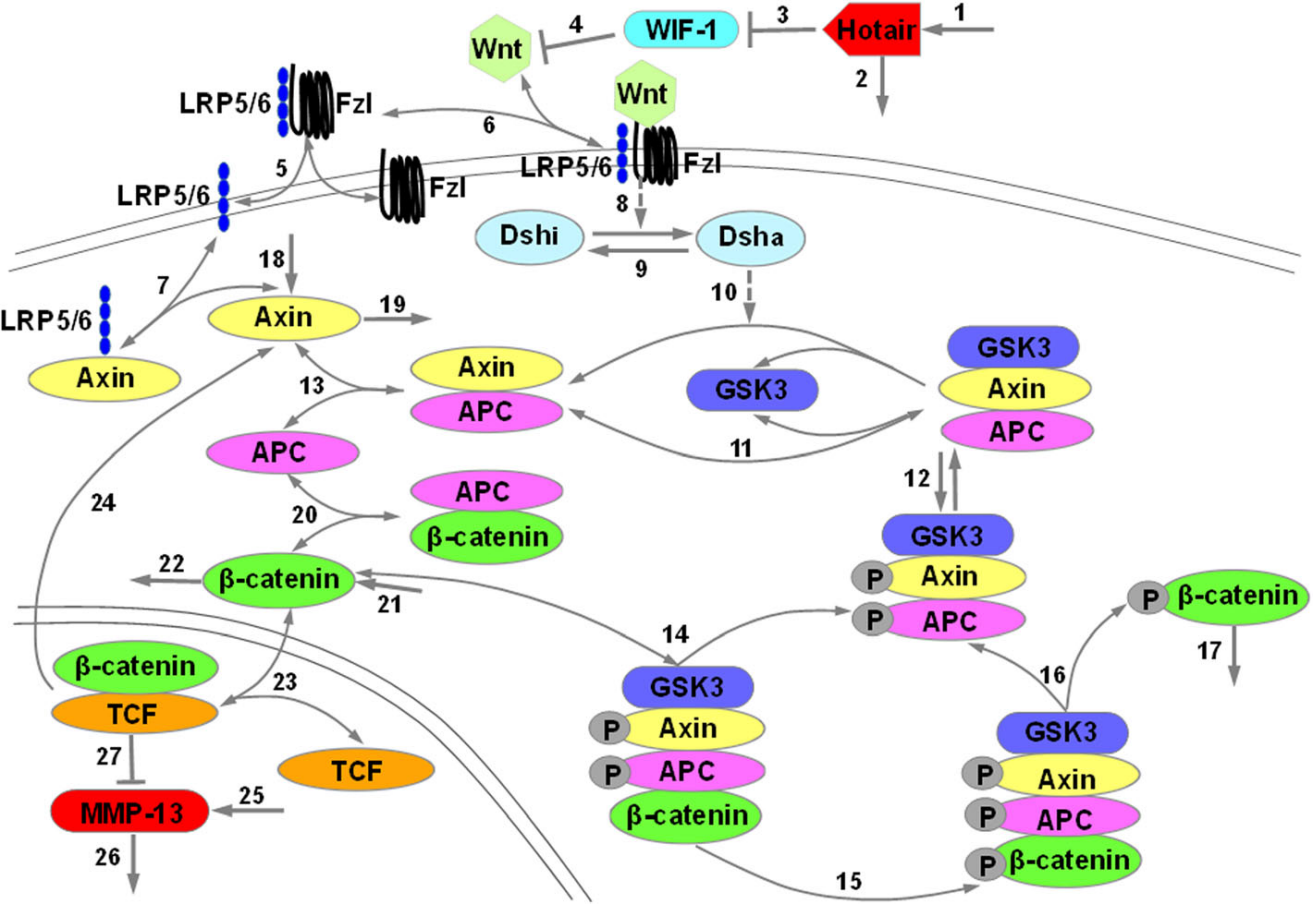
Reaction scheme for a model of the Hotair-mediated Wnt/β-catenin pathway

Model describing such systems is carried out presently by numerical integration of Eqs. (1)–(25), which uses a set of parameters with appropriate biological values (Table 1). Figure 1 shows a time series of the dynamic behavior of eight major components in HOTAIR-mediated Wnt/β-catenin pathway. With the regulation of HOTAIR (red curves), we can see that WIF-1 reaches steady state very quickly, i.e. in terms of minutes (Fig. 1a). This result is in a good agreement with the experimental observation that HOTAIR directly inhibited the expression of WIF-1 through increasing H3K27 trimethylation in the promoter region and then activated Wnt/β-catenin pathway [34]. Interestingly, we observed the oscillations of LRP5/6, GSK3, APC, Axin, TCF, β-catenin and MMP-13 within our model with a period close to 180 min, when they reach a steady state about 200 min later (Fig. 1b-h, red curves). This may suggest that Axin negative feedback loop is likely an important driving force in the system to produce oscillations, since Axin is a known suppresser of its own transcription which contributed to the formation of a negative feedback loop. As an essential component, the negative feedback loop is required for oscillation production in the dynamic model.

**Table 1:** Parameters and their default values for the model

Parameter	Process	Default Value	Reference
k_1_	Synthesis of HOTAIR	0.2 nM•min^− 1^	[23, 28]
k_2_	Degradation of HOTAIR	0.1 min^− 1^	[23, 28]
V_3_	Inhibition of WIF-1 synthesis	1 nM•min^− 1^	[23, 28]
k_+ 4_	Inhibition of Wnt synthesis	1 nM^− 1^•min^− 1^	[23, 28]
k_− 4_	Dissociation of WIF-1 from the [WIF-1.Wnt] complex	1 min^− 1^	[20]
k_+ 5_	Binding of LRP5/6 to Fzl to form [LRP5/6.Fzl]	0.1 nM^− 1^•min^− 1^	[30–32]
k_− 5_	Dissociation of [LRP5/6] into LRP5/6 and Fzl	1 min^− 1^	[30–32]
k_+ 6_	Binding of Wnt to [LRP5/6.Fzl] to form [Wnt.LRP5/6.Fzl]	1 nM^− 1^•min^− 1^	[30–32]
k_−6_	Dissociation of [Wnt.LRP5/6.Fzl] into Wnt and [LRP5/6. Fzl]	1 min^− 1^	[30–32]
k_+ 7_	Binding of LRP5/6 to Axin to form [LRP5/6.Axin]	1 nM^− 1^•min^− 1^	[30–32]
k_−7_	Dissociation of [LRP5/6.Axin] into LRP5/6 and Axin	1 min^− 1^	[30–32]
k_8_	Activation of Dsh	0.182 min^− 1^	[30–32]
k_9_	Deactivation of Dsh	0.0182 min^− 1^	[30–32]
k_10_	Dissociation of GSK3 from the destruction complex	0.05 nM^− 1^•min^− 1^	[30–32]
k_+ 11_	Binding of GSK3 to [Axin.APC] to form [GSK3. APC.Axin]	0.0909 nM^− 1^•min^− 1^	[30–32]
k_− 11_	Dissociation of [GSK3.APC.Axin] into GSK3 and [Axin.APC]	100 min^− 1^	[30–32]
k_+ 12_	Phosphorylation of Axin and APC	0.267 min^− 1^	[30–32]
k_− 12_	Dephosphorylation of Axin and APC	1 min^− 1^	[30–32]
k_+ 13_	Binding of APC to Axin to form [APC.Axin]	1 nM^− 1^•min^− 1^	[30–32]
k_− 13_	Dissociation of [APC.Axin] into APC and Axin	100 min^− 1^	[30–32]
k_+ 14_	Binding of β-catenin to [GSK3.APC*.Axin*] to form [GSK3.APC*.Axin*.β-catenin]	120 nM^− 1^•min^− 1^	[30–32]
k_− 14_	Dissociation of [GSK3.APC*.Axin*.β-catenin] into β-catenin and [GSK3.APC*.Axin*]	1 min^− 1^	[30–32]
k_15_	Phosphorylation of β-catenin	206 min^− 1^	[30–32]
k_16_	Dissociation of phosphorylated β-catenin	0.5 min^− 1^	[30–32]
k_17_	Degradation of phosphorylated β-catenin	0.417 min^− 1^	[30–32]
k_18_	Synthesis of Axin	8.22*10^− 5^ nM•min^− 1^	[30–32]
k_19_	Degradation of Axin	0.167 min^− 1^	[30–32]
k_+ 20_	Binding of APC to β-catenin to form [APC.β-catenin]	1 nM^− 1^•min^− 1^	[30–32]
k_− 20_	Dissociation of [APC.β-catenin] into APC and β-catenin	120 min^− 1^	[30–32]
k_21_	Synthesis of β-catenin	0.423 nM•min^− 1^	[30–32]
k_22_	Degradation of β-catenin	0.000257 min^− 1^	[30–32]
k_+ 23_	Binding of TCF to β-catenin to form [TCF.β-catenin]	2 nM^− 1^•min^− 1^	[30–32]
k_− 23_	Dissociation of [TCF.β-catenin] into TCF and β-catenin	20 min^− 1^	[30–32]
k_24_	Synthesis of Axin induced by [TCF.β-catenin]	0.02 min^− 1^	[30–32]
k_25_	Synthesis of MMP-13	0.1 nM•min^− 1^	[21]
k_26_	Degradation of MMP-13	0.1 min^− 1^	[21]
V_27_	Activation of MMP-13 synthesis	0.1 nM•min^− 1^	[21]
K_3_	Inhibition constant of WIF-1 by HOTAIR	0.1 nM	[23, 28]
K_8_	Activation constant of Dsh by Wnt	10 nM	[30, 31]
K_27_	Activation constant of MMP-13 by [TCF.β-catenin]	1 nM	[21]
m_3_	Degree of cooperativity of repression of WIF-1 expression by HOTAIR	4	[23, 28]
m_27_	Degree of cooperativity of activation of MMP-13 expression by [TCF.β-catenin]	4	[23, 28]
Wnt	Initial concentration of Wnt	10 nM	[30, 31, 33]
Fzl	Initial concentration of Fzl	10 nM	[30, 31, 33]
WIF-1	Initial concentration of WIF-1	10 nM	[30, 31, 33]
Dsh	Initial concentration of Dsh	100 nM	[30, 31, 33]
APC	Initial concentration of APC	100 nM	[30, 31, 33]
TCF	Initial concentration of TCF	15 nM	[30, 31, 33]
GSK3	Initial concentration of GSK3	50 nM	[30, 31, 33]

At the same time, the blue curves of Fig. 1 representing those situation of the system in the absence of HOTAIR uncover some other interesting information. In Fig. 1a, the expression of WIF-1 obviously increases linearly without the introduction of HOTAIR which is not in a steady state. This result reveals that HOTAIR is important in controlling the dynamic behavior of WIF-1, dysfunction of HOTAIR may lead to the disturbance of downstream signal transmission. However, only a subtle change (both frequency and amplitude) is showed in the oscillatory pattern of the LRP5/6, GSK3, APC, Axin, TCF, β-catenin and MMP-13 compared to the HOTAIR-mediated case (Fig. 1b-h, blue curves). Particularly, the oscillatory appearance of LRP5/6 changes more significantly, with the amplitude increased by about 30% (Fig. 1b). It may be because LRP5/6 is the upstream factor of the whole pathway and is closed to the disturbance from HOTAIR. Although the influence of HOTAIR regulation on the oscillatory pattern formation in cartilage damage is consistent with the fact that lncRNA plays a role in regulating the system in a relatively weak manner. HOTAIR is still essential in controlling the dynamics of the whole system, and the abnormal expression of HOTAIR may result in various diseases.

### Dynamic sensitivity analysis of the model

**Fig. 2:**
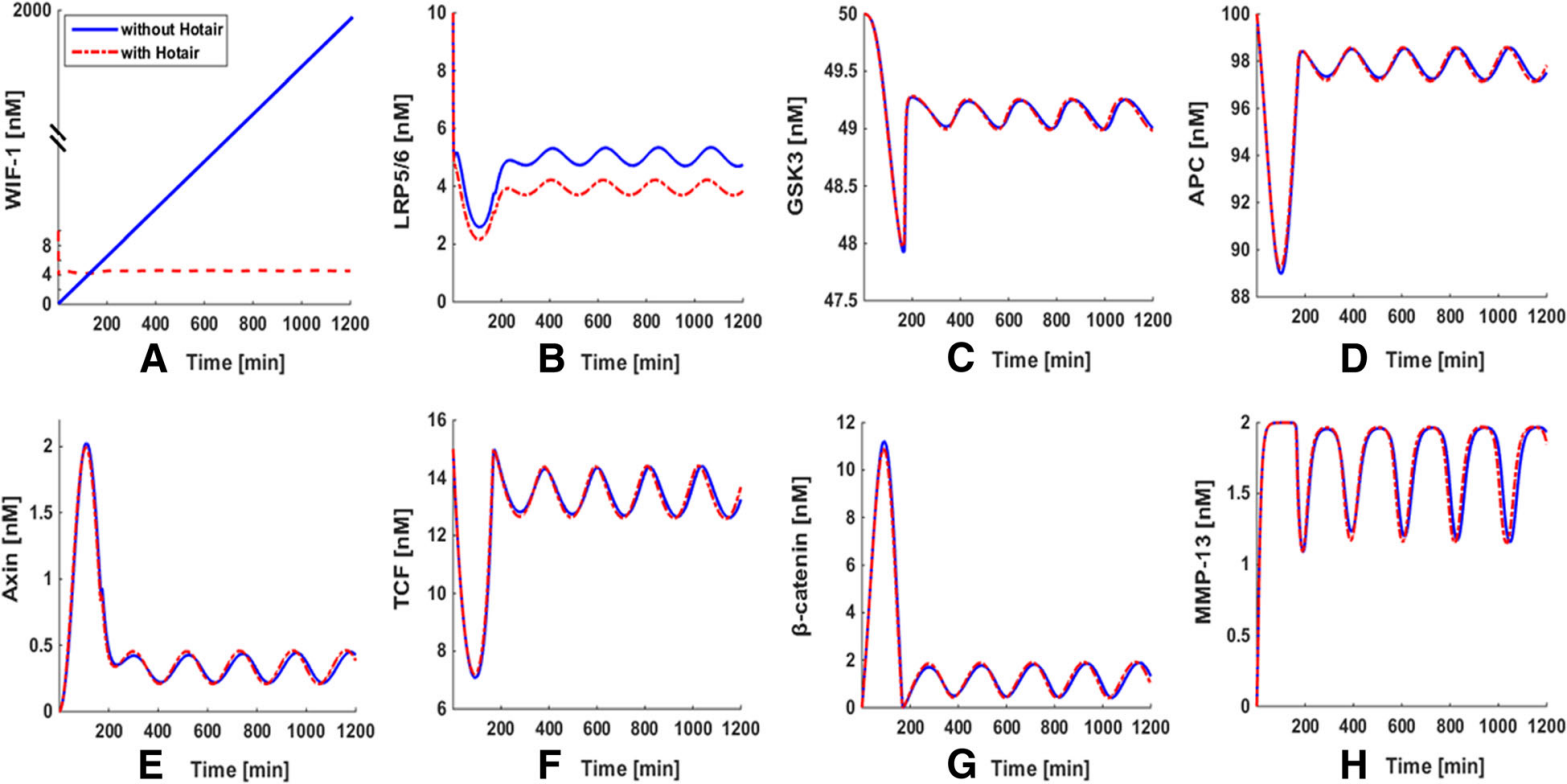
The temporal changes of the concentrations of key proteins with (red curves) or without (blue curves) the regulation of Hotair respectively. Time evolution of WIF-1, LRP5/6, GSK3, APC, Axin, TCF, β-catenin and MMP-13 (**a**-**h**)

In the present study, dynamic sensitivity analysis is carried out on a model of the HOTAIR-mediated Wnt/β-catenin pathway to determine how “sensitive” a model is to changes in the parameters causing changes in the dynamic behavior of the whole system. A sensitivity analysis for all parameters (Table 1) of our model was constructed, while a total of 1554 (42 rate constants × 37 reactions) local sensitivities were calculated and normalized with 144 scaled sensitivity absolute values (|S|). Finally, only 11 of the total 42 parameters have major influence on the whole pathway (|S|>2), where negative S represents the reaction output decreasing with the increasing rate constant. The heat maps of significant sensitivities of each reaction flux with respect to each parameter are picked out and shown in Fig. 2a, while those with weak or no influences (|S| < 2) on the model are ignored for clarity.

The number of reactions affected by the key parameters from sensitivity analysis are shown visually with a histogram in Fig. 2b. The rate parameter of Axin synthesis (k_24_) has the largest effect on the whole system which can affect 18 out of the total 37 reactions. While k_16_ (dissociation of phosphorylated β-catenin from Axin*_•_GSK3_•_APC*_•_β-catenin*) significantly affects 15 reactions, k_19_ (the degradation rate of Axin) affects 14 reactions, and k_11_ (binding rate of GSK3 to the APC_•_Axin complex), k_− 11_ (dissociation rate of GSK3 from the GSK3_•_APC_•_Axin complex), k_13_ (binding rate of APC to Axin), k_− 13_ (dissociation of APC from the APC_•_Axin complex) affect 13 reactions respectively. In summary, the influences of these parameters on the whole system obey the following order: k_24_ > k_16_ > k_19_ > k_11_ > k_− 11_ > k_13_ > k_− 13_. Interestingly, from these results we find that most of these parameters are all related to Axin. In particular, the most sensitive parameters are k_24_ and k_19_, all of which are directly relevant to the synthesis and degradation of Axin respectively. These observations indicate that the parameters related to Axin exhibit a marked dynamic effect in the whole model. It means that the dynamic behavior of HOTAIR-mediated Wnt/β-catenin pathway is highly sensitive to Axin, which may play a significant role in understanding the potential mechanism of cartilage damage. This implies that Axin may be a potential therapeutic intervention pointed to block cartilage damage.

### Effect of the variation of parameters on model dynamics

**Fig. 3:**
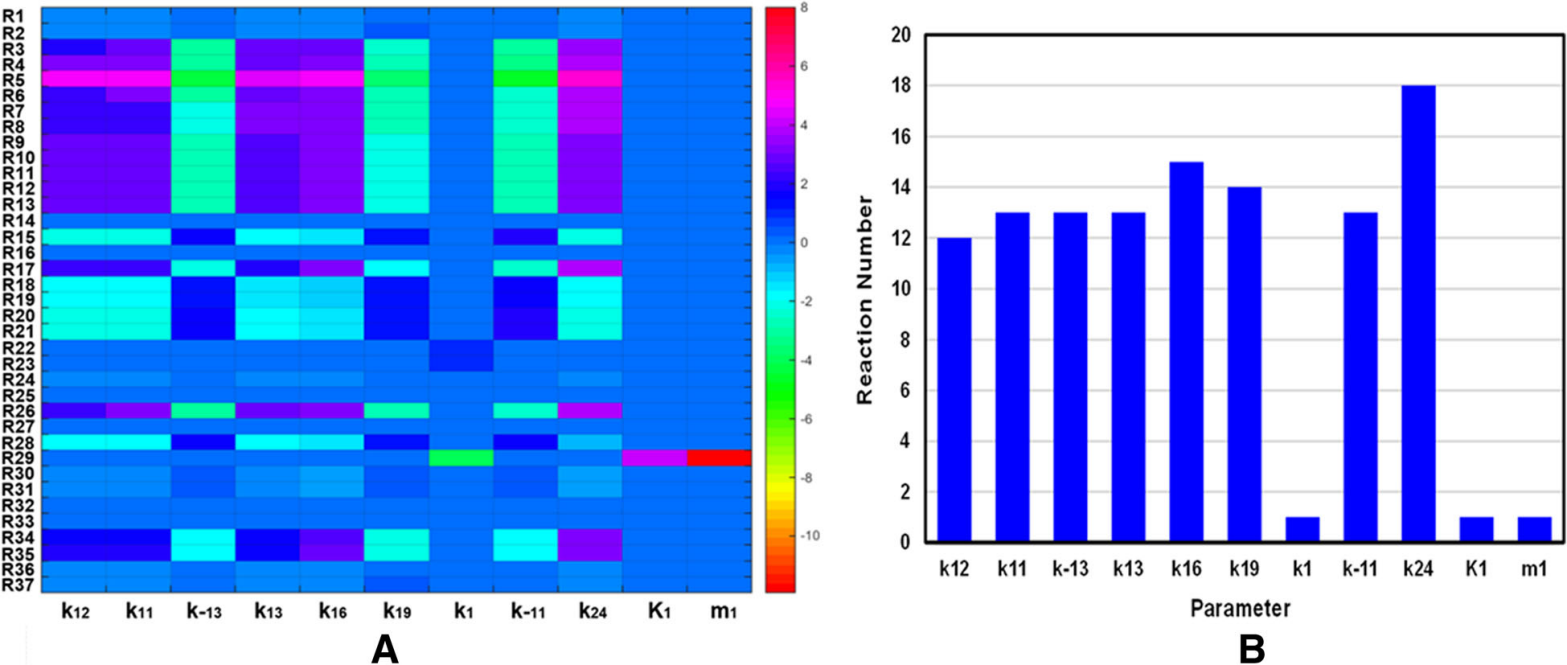
(**a**) The heat maps of local sensitivities of each reaction flux with respect to each parameter. (**b**) The number of reactions affected by the key parameters from sensitivity analysis

The sensitivity analysis shows that the system is the most sensitive to the variation of the synthesis rate of Axin (k_24_). Therefore, to examine how k_24_ exhibits the largest effects on our system, some explorations on variation of the kinetic parameter space were implemented presently. The kinetic parameter (k_24_) is 100 times increased or decreased compared to the ‘basal’ value of k_24_ = 0.02 nM•min^− 1^, when all other rate constants were kept fixed. The results are shown in Fig. 3, in which the blue, red and black curves represent the cases for k_24_ = 0.0002, 0.02 and 2 nM•min^− 1^, respectively.

By plotting the temporal trajectories of key components with k_24_ variations in the same panel, some interesting quantitative views are observed. No matter whether the reaction rate k_24_ has increased or decreased compared to the ‘basal’ value of k_24_ = 0.02 nM•min^− 1^, the oscillations of LRP5/6 (Fig. 3a), GSK3 (Fig. 3b), APC (Fig. 3c), Axin (Fig. 3d), TCF (Fig. 3e) and β-catenin (Fig. 3f) disappear and subsequently decline toward a steady state. It obviously shows that the key components of our system are very sensitive to k_24_ perturbation. These results are well consistent with the findings obtained from the sensitivity analysis, where k_24_ is the most sensitive parameter in our model. This implies that Axin may be considered a potential therapeutic intervention point for disturbing the dynamic system, so as to play a role in the treatment of cartilage damage.

### Effect of Axin on MMP-13 dynamics

**Fig. 4:**
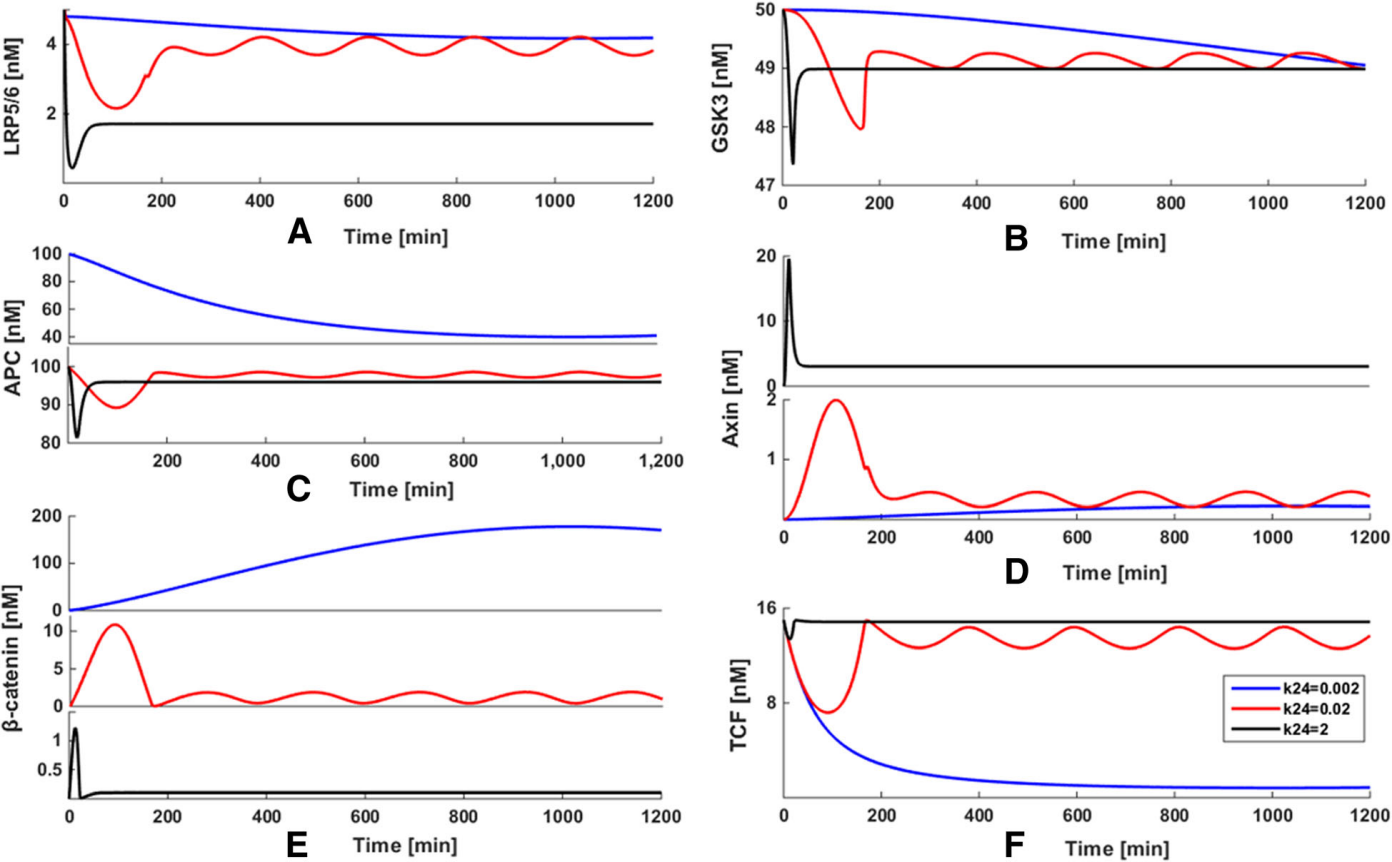
Effect of the variation of the synthesis rate of Axin (k24 nM•min-1) on the model

In order to investigate how the changes of the kinetic parameters related to Axin cause the effects on the MMP-13 dynamic behavior of our model, some attempts on parameter variations were developed for detailed analysis. The values of three most sensitive parameters k_16_, k_19_ and k_24_ were set to increase or decrease by 100 times, with all the other parameters fixed. The obtained results are shown in Fig. 4, in which the blue, red and black curves represent the variation of k_16_, k_19_ and k_24_, respectively.

As illustrated in Fig. 4a, we explored the variation of steady state level when k_16_ = 0.005, 0.5 (‘basal’ value) and 50 nM•min^− 1^, when keeping all other rate constants fixed. When k_16_ changes from 0.5 to 0.005 nM•min^− 1^ (blue curve) or 50 nM•min^− 1^ (black curve), the oscillatory behavior of MMP-13 appears in an extremely constant manner. In Fig. 4b, the value of k_19_ was changed as 0.00167, 0.167 and 16.7 nM•min^− 1^, with the values of other parameters were kept fixed. As k_19_ increases to 16.7 nM•min^− 1^ (black curve) or decreases to 0.00167 nM•min^− 1^ (blue curve), MMP-13 evolves to a steady state and no oscillations occur compared to the ‘basal’ value of k_19_ = 0.167 nM•min^− 1^. As seen in Fig. 4c, the value of k_24_ (the synthesis rate of Axin) was set to 0.0002, 0.02 (‘basal’ value) and 2 nM•min^− 1^.When k_24_ increases from 0.02 to 2 nM•min^− 1^ (black curve) or decreases from 0.02 to 0.0002 nM•min^− 1^(blue curve), the oscillations of MMP-13 disappear and subsequently reach a steady state. Since k_24_ is the synthesis rate of Axin that is a suppressor of Wnt/β-catenin pathway, the concentration of MMP-13 decreases when k_24_ rises to 2 nM•min^− 1^.

These results demonstrate that the parametersk_16_, k_19_ and k_24_ have significant effect on the oscillations of the deterministic system, which are well consistent with the findings obtained from the sensitivity analysis. More interestingly, compared with the variation in the amplitude of k_16_, the variation in the amplitude of MMP-13 changes significantly when k_19_ and k_24_ are whether enlarged or lessened by 100 times on the basis of the ‘basal’ values. This indicates that the parameters k_19_ and k_24_ with respect to the synthesis and degradation of Axin exert more marked effect on the dynamics of the model than k_16_. In other words, the mechanism of collagen type II degradation mediated by MMP-13 in articular cartilage is highly sensitive to Axin, which may a potential target for developing a therapeutic strategy toward blockade of cartilage damage and should be investigated further in an experimental setting. All the above results not only show the significant roles of parameter perturbation for the whole system, but also prove the reliability of dynamic sensitivity analysis, which might provide a promising strategy for the prediction of potential therapeutic intervention point for the prevention of cartilage damage.

### The experiment evaluation of therapeutic intervention point

**Fig. 5:**
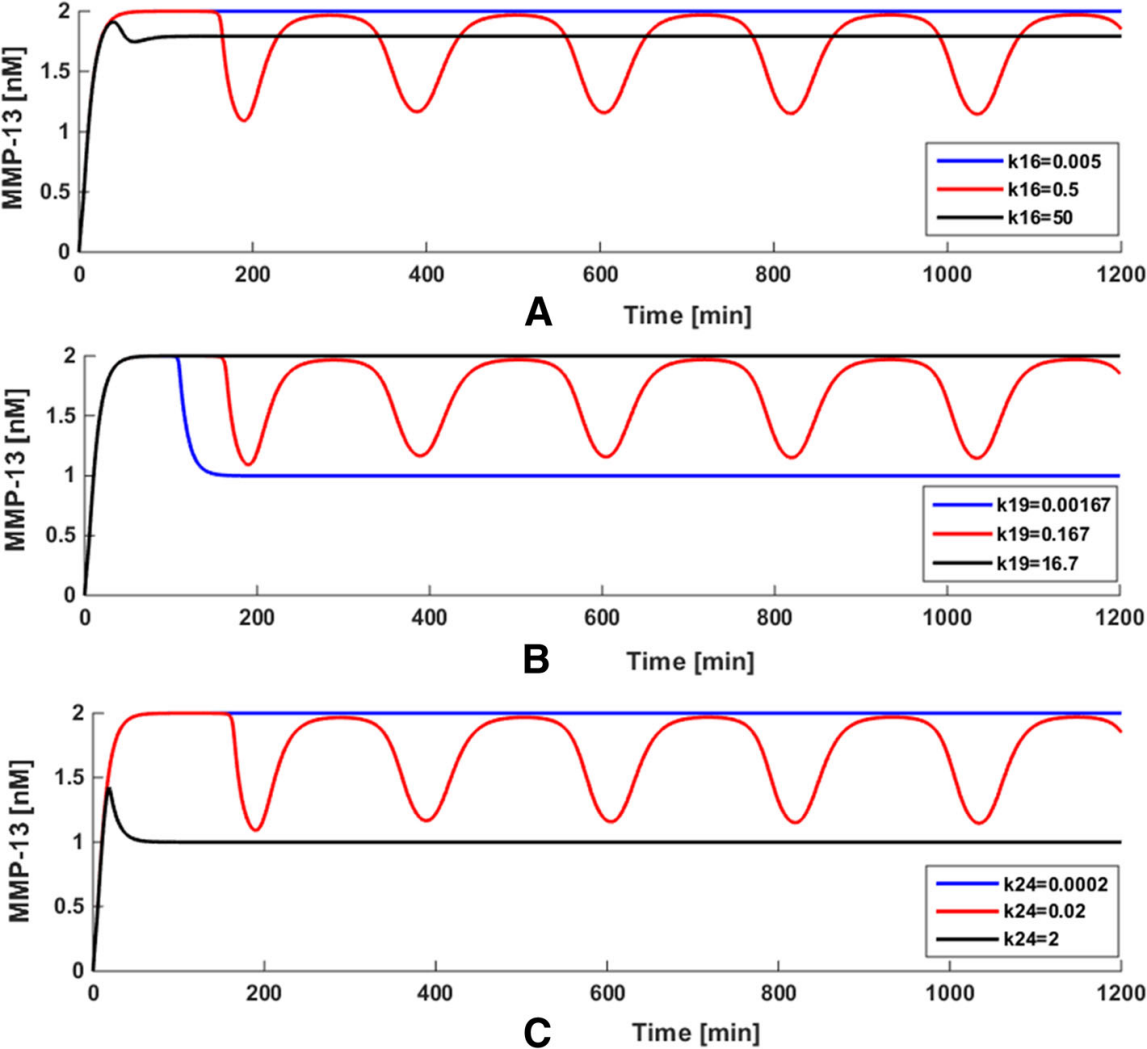
Temporal behavior of MMP-13 in the model with the perturbation of parameters k16, k19 and k24

Previous studies indicate that Wnt signaling induces Axin2 expression in different cell types. To determine if Wnt 3a induce Axin2 expression, we researched Axin2 mRNA expression in Wnt 3a-treated chondrocytes. The expression of Axin2 gene was rapidly induced by Wnt3a, while IL-1β had no effect on Axin2 expression (Fig. 5a). Then, to observe whether increased Axin2 could inhibit the expression of MMP-13 in Hc-a stimulated with IL-1β, we measured the MMP-13 expression in supernatant of Wnt 3a-treated Ha-c with or without IL-1β stimulation. As shown in Fig. 5b, IL-1β potently induced MMP-13 production in Ha-c, and increased Axin2 could remarkably inhibit the production of MMP-13. The obtained experimental results were in agreement with our prediction, suggesting that our method was reasonable and accurate to assess the therapeutic intervention point for the prevention of cartilage damage.

## Discussion

In this paper, a system-theoretic approach by employing a chemical kinetic reactions approach based on ODEs is introduced to construct the model of HOTAIR-mediated Wnt/β-catenin pathway which is used not only to quantitatively describe the dynamic mechanism of key components in the model, but also to predict the possible interventions for preventing cartilage damage.

Firstly, using the kinetic parameters with appropriate biological values, the modeling work was carried out by numerical integration of 25 ODEs. Our model reveals the existence of biological oscillatory behavior through modeling the time series of the dynamic behavior of key signaling molecules in the system. The reasonability of this model has demonstrated by clearly oscillations which were due to the fact that Axin is a likely component of the negative feedback loop, which is activated by β-catenin and then is degraded by a complex of Axin, APC and GSK3. In addition, we also point out the role of HOTAIR on the regulation of Wnt/β-catenin pathway. Although the effect of HOTAIR regulation on the oscillatory pattern formation in the system is weak, HOTAIR plays a significant role in maintaining the equilibrium state and activates Wnt/β-catenin pathway by inhibiting WIF-1 expression. Secondly, the dynamic sensitivity analysis was performed to investigate the oscillatory dynamic behavior of the model with changes in parameters to explore which parameters were the most crucial ones impacting the whole system. The obtained result suggested that the parameters with respect to the synthesis and degradation of Axin had the largest impact on our model which implied the system is highly sensitive to Axin. Thirdly, some explorations on variation of the parameter space were carried out for detailed analysis to examine how the effect of Axin causes changes in the dynamic behavior of our model. The result was well consistent with the finding obtained from the dynamic sensitivity analysis. Finally, the experiment is applied to validate the reasonability of our strategy, thus to provide a credible method to assess therapeutic intervention points for the prevention of cartilage damage. The experimental observations were in agreement with our prediction, confirming that our method is reliable for developing a tractable therapeutic target toward blockade of cartilage damage.

## Conclusion

In conclusion, it is urgent to improve our understanding of the molecular mechanisms involved in cartilage breakdown and to develop new therapeutic intervention points. Not surprisingly, systems modeling approaches have become increasingly recognized as a tool to explore these problems as the complement of experimental work. The successful development of our strategy will not only be helpful for deep understanding of the underlying dynamic molecular mechanism of HOTAIR-mediated Wnt/β-catenin signaling pathway, but also provide a promising idea to predict the potential intervention for further experimental verification, so as to contribute to the prevention of the various pathological conditions related to cartilage damage.

In this work, all the programs were implemented on a Dell workstation and the CPU running time is obtained on Intel Xeon E5–1650 v3 6-Core 3.5GHz 15MB CPU and 8 GB RAM. An ODE solver in MATLAB was used to carry out our deterministic simulation. Accuracy is controlled by setting an absolute tolerance of 10–8 applied to all the variables. The challenge for future work will be a focus on the improvement of accuracy and comprehensiveness in the model, since lack of reliable biological experimental data and parameters available for model construction limits the restoration of the real biological system.

## Methods

### Construction of the model of the HOTAIR-mediated Wnt/β-catenin pathway

**Fig. 6:**
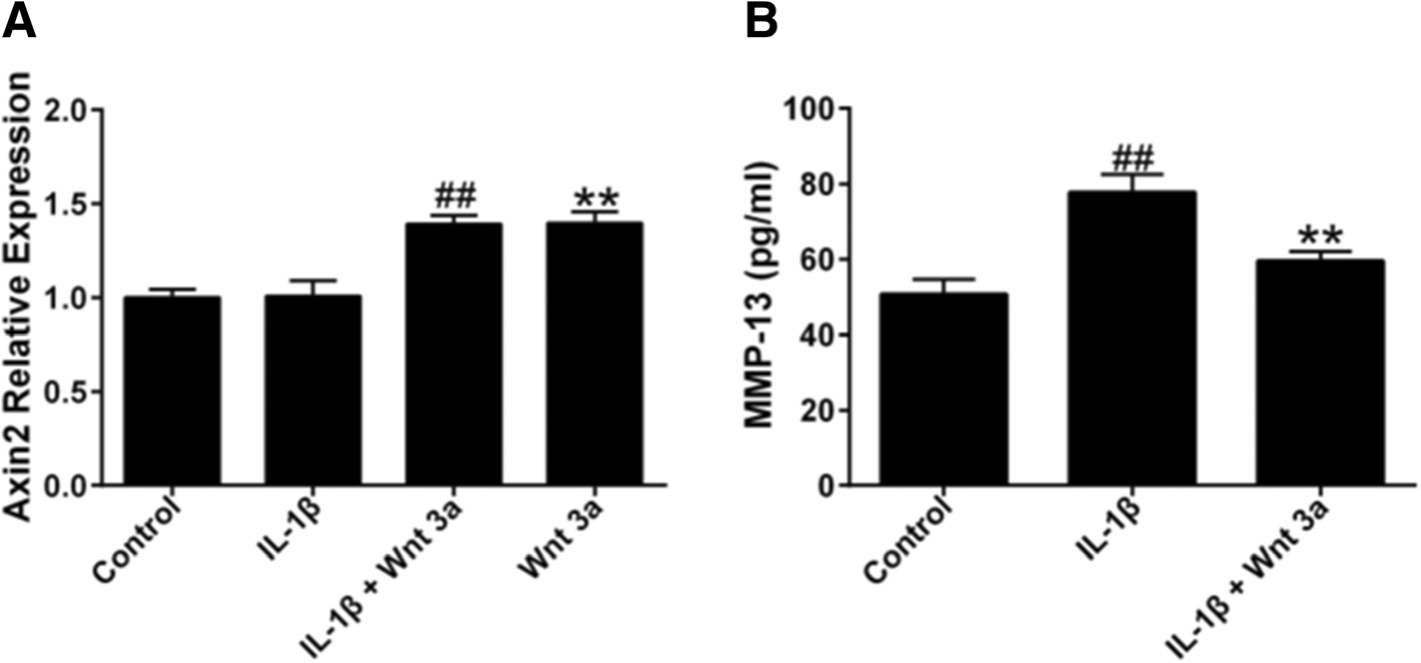
Effect of Axin on MMP-13 Expression in IL-1β-Stimulated Hc-a. The Ha-c cells were plated into a 6-well culture plate at 2 × 105 cells/well and then allowed to adhere overnight. After incubation, the cultured cells were treated with 100 ng/ml Wnt 3a in triplicate with or without 10 ng/ml IL-1β for 24 h. After 24 h, the cells were collected for the measurement of Axin with RT-PCR (**a**), and the cell supernatant was collected for MMP-13 detection by using ELISA (**b**). ## *P* < 0.01 compared with Control group, ** *P* < 0.01 compared with IL-1β group

In this work, a mathematical model for HOTAIR-mediated Wnt/β-catenin dynamic network regulating MMP-13 was developed by employing a chemical kinetic reactions approach based on ODEs. The HOTAIR-mediated Wnt/β-catenin pathway is defined in terms of a number of signaling molecules and reaction steps, which forms the basis for the mathematical model of the pathway. It is well know that β-catenin is the central and essential factor of Wnt/β-catenin pathway, which is regulated by the cytoplasmic destruction complex formed by Axin, GSK-3 and APC [35]. Hence the accumulation of β-catenin can interact with the transcription factor TCF/LEF and then regulate target gene expression [36]. Therefore, although many signaling molecules are involved in the Wnt/β-catenin pathway, we have paid attention on the above-mentioned core components considered to be necessary for regulating a Wnt signal in most cases. These key signaling molecules include HOTAIR, WIF-1, Wnt, Dishevelled (Dsh), GSK3, APC, Axin, β-catenin, TCF and MMP-13. The model is based on the reaction scheme shown in Fig. 6.

The model is initiated based on the fact that lncRNA HOTAIR activated the Wnt signal by directly repressing the expression of WIF-1 (steps 3, 4) [22]. In the case of the existence of Wnt signal, Wnt binds to the cell surface receptor Frizzled in combination with the low-density lipoprotein receptor protein (LRP5/6) 5 or 6 (steps 5,6), then Dsh is activated (step 8) and converts Dsh from inactive (Dsh_i_) to active form (Dsh_a_). At this point, GSK3 is dissociated from a so called destruction complex (step 10), which is the center of this model that compose of the phosphorylated forms of the scaffolds APC, Axin and GSK3 (steps 14, 15) [35]. The function of this complex is to phosphorylate β-catenin and then ubiquitination-dependent degraded by the proteasome (steps 16, 17) [36]. The phosphorylation and dephosphorylation of the two scaffolds (APC and Axin) by GSK3 and a phosphatase are represented in Steps 11 and 12 respectively. A negative feedback is introduced into the model to represent well-established observation that β-catenin/TCF complex directly induces the synthesis of Axin (step 24). In addition, the model also takes into account the deactivation of Dsh (step 9), the syntheses of all proteins (steps 1, 18, 21, 25), their degradations (steps 2, 17, 19, 22, 26), and reversible binding of Axin to LRP5/6 (step 7), Axin to APC (step 13), β-catenin to the transcription factor APC (step 20) and β-catenin to TCF (step 23), as well as the transcriptional regulation of target MMP-13 by β-catenin/TCF complex (step 27) [21].

The reaction steps of HOTAIR-mediated Wnt/β-catenin pathway are modelled using standard reaction kinetics, leading to a set of ODEs based on the law of mass action and Hill-type function, which are the mathematical methods that explain and predict the behaviours of the molecules in dynamic equilibrium. The time evolution of the model is governed by a system of 25 kinetic equations. The ODEs of the dynamic model are presented as follows:

1 $$ \frac{d\left[ Hotair\right]}{dt}=k1-k2\left[ Hotair\right] $$

2 $$ \frac{d\left[ WIF\_1\right]}{dt}=\frac{V3\bullet K{3}^{m3}}{K{3}^{m3}+{\left[ Hotair\right]}^{m3}}+k\hbox{-} 4\left[ WIF\_1.\kern0.5em Wnt\right]-k4\left[ WIF\_1\right]\left[ Wnt\right] $$

3 $$ {\displaystyle \begin{array}{l}\frac{d\left[ Wnt\right]}{dt}=k\hbox{-} 4\left[ WIF\_1.\kern0.5em Wnt\right]-k4\left[ WIF\_1\right]\left[ Wnt\right]\\ {}\kern4em +k\hbox{-} 6\left[ Wnt\kern0.5em .\kern0.5em Fzl\kern0.5em .\kern0.5em LRP5/6\right]-k6\left[ Wnt\right]\left[ Fzl\kern0.5em .\kern0.5em LRP5/6\right]\end{array}} $$

4 $$ \frac{d\left[ WIF\_1.\kern0.5em Wnt\right]}{dt}=k4\left[ WIF\_1\right]\left[ Wnt\right]-k\hbox{-} 4\left[ WIF\_1.\kern0.5em Wnt\right] $$

5 $$ {\displaystyle \begin{array}{l}\frac{d\left[ LRP5/6\kern0.5em \right]}{dt}=k\hbox{-} 5\left[ LRP5/6\kern0.5em .\kern0.5em Fzl\right]-k5\left[ LRP5/6\right]\left[\kern0.5em Fzl\right]\\ {}\kern6em +k\hbox{-} 7\left[ Axin\kern0.5em .\kern0.5em LRP5/6\right]-k7\left[\kern0.5em Axin\right]\left[ LRP5/6\right]\end{array}} $$

6 $$ \frac{d\left[ Fzl\kern0.5em \right]}{dt}=k\hbox{-} 5\left[ Fzl\kern0.5em .\kern0.5em LRP5/6\right]-k5\left[\kern0.5em Fzl\right]\left[ LRP5/6\right] $$

7 $$ {\displaystyle \begin{array}{l}\frac{d\left[ Fzl\kern0.5em .\kern0.5em LRP5/6\kern0.5em \right]}{dt}=k5\left[\kern0.5em Fzl\right]\left[ LRP5/6\right]-k\hbox{-} 5\left[ Fzl\kern0.5em .\kern0.5em LRP5/6\right]\\ {}\kern8em +k\hbox{-} 6\left[ Wnt.\kern0.5em Fzl\kern0.5em .\kern0.5em LRP5/6\right]-k6\left[ Wnt\right]\left[ Fzl\kern0.5em .\kern0.5em LRP5/6\right]\end{array}} $$

8 $$ \frac{d\left[ Wnt.\kern0.5em Fzl\kern0.5em .\kern0.5em LRP5/6\right]}{dt}=k6\left[ Wnt\right]\left[ Fzl\kern0.5em .\kern0.5em LRP5/6\right]-k\hbox{-} 6\left[ Wnt.\kern0.5em Fzl\kern0.5em .\kern0.5em LRP5/6\right] $$

9 $$ \frac{d\left[ Axin\kern0.5em .\kern0.5em LRP5/6\kern0.5em \right]}{dt}=k7\left[\kern0.5em Axin\right]\left[ LRP5/6\right]-k\hbox{-} 7\left[ Axin\kern0.5em .\kern0.5em LRP5/6\right] $$

10 $$ {\displaystyle \begin{array}{l}\frac{d\left[ Axin\right]}{dt}=k18-k19\left[ Axin\right]+k\hbox{-} 13\left[ APC\kern0.5em .\kern0.5em Axin\right]-k13\left[ APC\right]\left[ Axin\right]\\ {}\kern4.25em +k\hbox{-} 7\left[ Axin\kern0.5em .\kern0.5em LRP5/6\right]-k7\left[\kern0.5em Axin\right]\left[ LRP5/6\right]\\ {}\kern4.25em +k24\left(\left[\beta - catenin\kern0.6em .\kern0.5em TCF\right]+\left[\beta - catenin\right]\right)\end{array}} $$

11 $$ \frac{d\left[ Dsh i\right]}{dt}=k9\left[ Dsh\mathrm{a}\right]-\frac{k8\left[ Wnt.\kern0.5em Fzl\kern0.5em .\kern0.5em LRP5/6\right]\left[ Dsh\mathrm{i}\right]}{K8+\left[ Dsh\mathrm{i}\right]} $$

12 $$ \frac{d\left[ Dsh a\right]}{dt}=\frac{k8\left[ Wnt.\kern0.5em Fzl\kern0.5em .\kern0.5em LRP5/6\right]\left[ Dsh\mathrm{i}\right]}{K8+\left[ Dsh\mathrm{i}\right]}-k9\left[ Dsh\mathrm{a}\right] $$

13 $$ {\displaystyle \begin{array}{l}\frac{d\left[ APC/ Axin/ GSK3\right]}{dt}=-k10\left[ APC.\kern0.5em Axin\kern0.5em .\kern0.5em GSK3\right]\left[ Dsha\right]\\ {}\kern9.5em -k\hbox{-} 11\left[ APC\kern0.5em .\kern0.5em Axin\kern0.5em .\kern0.5em GSK3\right]\\ {}\kern17.5em -k12\left[ APC\kern0.5em .\kern0.5em Axin\kern0.5em .\kern0.5em GSK3\right]\\ {}\kern9.75em +k\hbox{-} 12\left[ APC\ast \kern0.5em .\kern0.5em Axin\ast \kern0.5em .\kern0.5em GSK3\right]\\ {}\kern17.5em +k11\left[ GSK3\right]\left[ APC\kern0.5em .\kern0.5em Axin\right]\end{array}} $$

14 $$ {\displaystyle \begin{array}{l}\frac{d\left[ APC\ast / Axin\ast / GSK3\right]}{dt}=k16\left[\beta - catenin\ast \kern0.5em .\kern0.5em APC\ast \kern0.5em .\kern0.5em Axin\ast \kern0.5em .\kern0.5em GSK3\right]\\ {}\kern17.5em +k12\left[ APC\kern0.5em .\kern0.5em Axin\kern0.5em .\kern0.5em GSK3\right]\\ {}\kern9.5em -k\hbox{-} 12\left[ APC\ast \kern0.5em .\kern0.5em Axin\ast \kern0.5em .\kern0.5em GSK3\right]\\ {}\kern17.5em +k\hbox{-} 14\left[\beta - catenin.\kern0.5em APC\ast \kern0.5em .\kern0.5em Axin\ast \kern0.5em .\kern0.5em GSK3\right]\\ {}\kern17.5em -k14\left[ APC\ast \kern0.5em .\kern0.5em Axin\ast \kern0.5em .\kern0.5em GSK3\right]\left[\beta - catenin\right]\end{array}} $$

15 $$ {\displaystyle \begin{array}{l}\frac{d\left[ GSK3\right]}{dt}=k10\left[ APC.\kern0.5em Axin\kern0.5em .\kern0.5em GSK3\right]\left[ Dsha\right]\\ {}\kern4.75em +k\hbox{-} 11\left[ APC\kern0.5em .\kern0.5em Axin\kern0.5em .\kern0.5em GSK3\right]\\ {}\kern4.5em -k11\left[ GSK3\right]\left[ APC\kern0.5em .\kern0.5em Axin\kern0.5em \right]\end{array}} $$

16 $$ {\displaystyle \begin{array}{l}\frac{d\left[ APC\kern0.5em .\kern0.5em Axin\right]}{dt}=k10\left[ APC\kern0.5em .\kern0.5em Axin\kern0.5em .\kern0.5em GSK3\right]\left[ Dsha\right]\\ {}\kern6.75em +k\hbox{-} 11\left[ APC\kern0.5em .\kern0.5em Axin\kern0.5em .\kern0.5em GSK3\right]-k11\left[ GSK3\right]\left[ APC\kern0.5em . Axin\right]\\ {}\kern7em -k\hbox{-} 13\left[ APC\kern0.5em .\kern0.5em Axin\right]+k13\left[ APC\right]\left[ Axin\right]\end{array}} $$

17 $$ {\displaystyle \begin{array}{l}\frac{d\left[ APC\right]}{dt}=-k20\left[ APC\right]\left[\beta - catenin\right]+k\hbox{-} 20\left[\beta - catenin\kern0.5em . APC\right]\\ {}\kern8em +k\hbox{-} 13\left[ APC\kern0.5em .\kern0.5em Axin\right]-k13\left[ APC\right]\left[ Axin\right]\end{array}} $$

18 $$ {\displaystyle \begin{array}{l}\frac{d\left[\beta - catenin/ APC\ast / Axin\ast / GSK3\right]}{dt}=-k15\left[\beta - catenin\kern0.5em .\kern0.5em APC\ast \kern0.5em .\kern0.5em Axin\ast \kern0.5em .\kern0.5em GSK3\right]\\ {}\kern26.5em -k\hbox{-} 14\left[\beta - catenin.\kern0.5em APC\ast \kern0.5em .\kern0.5em Axin\ast \kern0.5em .\kern0.5em GSK3\right]\\ {}\kern26.5em +k14\left[ APC\ast \kern0.5em .\kern0.5em Axin\ast \kern0.5em .\kern0.5em GSK3\right]\left[\beta - catenin\right]\end{array}} $$

19 $$ {\displaystyle \begin{array}{l}\frac{d\left[\beta - catenin\ast / APC\ast / Axin\ast / GSK3\right]}{dt}=k15\left[\beta - catenin\kern0.5em .\kern0.5em APC\ast \kern0.5em .\kern0.5em Axin\ast \kern0.5em .\kern0.5em GSK3\right]\\ {}\kern16.5em -k16\left[\beta - catenin\ast \kern0.5em .\kern0.5em APC\ast \kern0.5em .\kern0.5em Axin\ast \kern0.5em .\kern0.5em GSK3\right]\end{array}} $$

20 $$ \frac{d\left[\beta - catenin\ast \right]}{dt}=k16\left[\beta - catenin\ast \kern0.5em .\kern0.5em APC\ast \kern0.5em .\kern0.5em Axin\ast \kern0.5em .\kern0.5em GSK3\right]-k17\left[\beta - catenin\ast \right] $$

21 $$ {\displaystyle \begin{array}{l}\frac{d\left[\beta - catenin\right]}{dt}=k21-k22\left[\beta - catenin\right]-k23\left[\beta - catenin\right]\left[ TCF\right]\\ {}\kern7em +k\hbox{-} 23\left[\beta - catenin\kern0.6em .\kern0.5em TCF\right]-k20\left[\beta - catenin\right]\left[ APC\right]\\ {}\kern7em +k\hbox{-} 20\left[\beta - catenin\kern0.5em .\kern0.5em APC\right]\\ {}\kern7em +k\hbox{-} 14\left[\beta - catenin\kern0.5em .\kern0.5em APC\ast \kern0.5em .\kern0.5em Axin\ast \kern0.5em .\kern0.5em GSK3\right]\\ {}\kern12.5em -k14\left[ APC\ast \kern0.5em .\kern0.5em Axin\ast \kern0.5em .\kern0.5em GSK3\right]\left[\beta - catenin\kern0.5em \right]\end{array}} $$

22 $$ \frac{d\left[ TCF\right]}{dt}=-k23\left[\beta - catenin\right]\left[ TCF\right]+k\hbox{-} 23\left[\beta - catenin\kern0.6em .\kern0.5em TCF\right] $$

23 $$ \frac{d\left[\beta - catenin/ TCF\right]}{dt}=k23\left[\beta - catenin\right]\left[ TCF\right]-k\hbox{-} 23\left[\beta - catenin\kern0.6em .\kern0.5em TCF\right] $$

24 $$ \frac{d\left[\beta - catenin/ APC\right]}{dt}=k20\left[ APC\right]\left[\beta - catenin\right]-k\hbox{-} 20\left[\beta - catenin\kern0.5em .\kern0.5em APC\right] $$

25 $$ \frac{d\left[ MMP-13\right]}{dt}=k25+\frac{V27\bullet K{27}^{m27}}{K{27}^{m27}+{\left[\beta - catenin\kern0.6em .\kern0.5em TCF\right]}^{m27}}-k26\left[ MMP-13\right] $$

The kinetic parameter values used in 25 ODEs were chosen within a reasonable physiological range where available. Semi-arbitrary choice of parameter values were explored in order to best reproduce the essential responses associated with experimental measurements. All parameter values were obtained and estimated according to their relevant references. The parameter values and their biological explanations are given in Table 1.

### Dynamic sensitivity analysis

Dynamic mathematical models usually involve a large number of physicochemical parameters to describe biological models, such as gene regulation, signaling and metabolic networks. It is necessary to investigate how the model changes with perturbations in the parameter values, since any small changes in the value of the parameters may drastically affect the output of system [37]. Sensitivity analysis (*S*_*A*_) is a useful tool to understand the behavior of dynamic systems, which characterizes the influence of sensitive the parameter changes on the outputs of the system.

A dynamic sensitivity value characterizes the response of state variable (output) to a parameter (input) variation on model at any time. Thus, the sensitivity of the model to the output Y with respect to a single parameter X_i_ can be calculated as:

26 $$ {S}_A=\frac{\partial Y}{\partial X\mathrm{i}} $$

The local sensitivity index (*S*_*A*_) is defined as the relative change in state variable Y (output) divided by the relative change in the parameter X_i_. It examines the effect of each parameter independently corresponding to the variable at one specific moment. The results are normalized in order to obtain the dimensionless scaled sensitivity of an input factor for plotting the visual presentation drawing.

### Experimental verification

#### Culture of human articular chondrocytes

Human articular chondrocytes (Ha-c) were purchased from ScienCell Research Laboratories (Carlsbad, CA, USA) and maintained in Dulbecco’s modified Eagle’s medium (DMEM) containing 10% fetal bovine serum (FBS) (Gibco BRL, Gaithersburg, MD, USA). For the experiments, the Ha-c cells were plated into a 6-well culture plate at 2 × 10^5^ cells/well and then allowed to adhere overnight. After incubation, the cultured cells were treated with 100 ng/ml Wnt 3a in triplicate with or without 10 ng/ml IL-1β for 24 h at 37 °C in a humidified 5% CO_2_ incubator. After 24 h, the cell supernatant and cells were collected for detection.

#### Measurement of MMP-13

The MMP-13 level in cell supernatant was detected using MMP-13 ELISA kit (Abcam, Cambridge, UK) according to the manufacturer’s protocol.

#### Real time-PCR

The total RNA of Ha-c cells was isolated using TRIzol Reagent (Invitrogen, Carlsbad, CA, USA) following the manufacturer’s instructions. After isolating the total RNA from Ha-c cells, 1 μg was reverse transcribed using QuantiTect Reverse Transcription Kit (QIAGEN K.K., Tokyo, Japan). The specific transcripts were optimized by quantitative real-time PCR with QuantiTect SYBR Green PCR Kit (QIAGEN K.K.) and ABI 7500 real-time PCR system (Applied Biosystems, Foster, CA, USA) were used to analyze results. Gene-specific primers used were as follows: Axin2 (GCTTGGAGACAATGCTGTTG as reverse and GAGGGAGAATGCGTGG ATA as forward), GAPDH (AGGGGCCATCCACGTCTTC as reverse and AGAAGGCTGGGGCTCATTTG as forward). Real-time PCR performed as 40 cycles for 30 s at 95 °C, 5 s at 95 °C, 30 s at 60 °C, 15 s at 95 °C, 1 min at 60 °C and 15 s at 95 °C. The date were calculated using the ∆∆Ct algorithm and were normalized to GAPDH expression.

## Data Availability

All data generated or analysed during this study are included in this published article.

## Abbreviations

APC: Adenomatous polyposis coli
DMEM: Dulbecco’s modified Eagle’s medium
Dsh: Dishevelled
ECM: Extracellular matrix
FBS: Fetal bovine serum
GSK3: Glycogen synthase kinase 3
LncRNA: Long non-coding RNA
MMPs: Matrix metalloproteinases
OA: Osteoarthritis
ODEs: Ordinary differential equations
RA: Rheumatoid arthritis
